# Childhood leukaemia incidence and the population mixing hypothesis in US SEER data

**DOI:** 10.1038/sj.bjc.6601734

**Published:** 2004-03-30

**Authors:** D Wartenberg, D Schneider, S Brown

**Affiliations:** 1 UMDNJ-Robert Wood Johnson Medical School, The Cancer Institute of New Jersey, New Brunswick, NJ, 170 Frelinghuysen Road, Piscataway, NJ 08854, USA; 2 Rutgers University, The State University of New Jersey, The Cancer Institute of New Jersey, 33 Livingston Avenue, Suite 100, New Brunswick, NJ 08901-1958, USA; 3 College of Public Health, University of Arizona, Tucson, AZ, USA

**Keywords:** childhood cancer, leukaemia, population mixing, SEER

## Abstract

We evaluated the infectious aetiology hypothesis of childhood leukaemia that rapid population influx into rural areas is associated with increased risk. Using data from the US SEER program, we found that in changes in rural county population sizes from 1980 to 1989 were associated with incidence rates for childhood acute lymphocytic leukaemia (ALL). The observed associations were strongest among children 0–4 years of age, born in the same state as diagnosis, in extremely rural counties, and when counties adjacent to nonrural counties were excluded. Similar analyses for brain and central nervous system (CNS) cancer in children, a disease less linked to this infectious hypothesis, provide evidence against methodologic bias. Similar evaluations for other decades were not meaningful due to limited sample sizes and, perhaps, increased population mobility.

Leukaemias represent 25% of childhood cancers, approximately 3250 cases per year in the US (Smith *et al*, 1999). While childhood leukaemia can be caused by ionising radiation, certain chemotherapeutic agents and specific genetic disorders (e.g. Down's syndrome), and may also be associated with other factors (e.g., parental occupation, magnetic fields), the majority of cases remain unexplained (Kipen and Wartenberg, 1994; Greaves, 1997,^2002^; Smith *et al*, 1999). Even though unusual clustering of cases has often been seen (Boyle *et al*, 1996), their investigation has not led to a better understanding of their causes.

In 1988, seeking to explain a childhood leukaemia cluster in Seascale, the community adjacent to the Sellafield nuclear power station in England, and a comparable situation in Scotland, the Population Mixing Hypothesis was proposed (Kinlen, 1988). This built on the view that in most cases childhood leukaemia is a rare response to a common but unidentified infection and held that a localised epidemic of this underlying infection might occur, as epidemics of other such diseases have occurred, when a large group of individuals (many with urban backgrounds and therefore exposed to a wide variety of infections) moved into a sparsely populated area in which a substantial proportion of the population have not been so exposed and were therefore susceptible to infection. This paper investigates whether patterns consistent with the Population Mixing Hypothesis are discernable in data from the US National Cancer Institute's Surveillance, Epidemiology and End Results (SEER) Program (SEER, 2001).

## SUBJECTS AND METHODS

We examined incidence rates for acute lymphocytic leukaemia (ALL) in children as a function of change in population size. To control for possible methodologic or reporting bias, we conducted identical analyses for brain and central nervous system (CNS) cancers in children. We considered assessing patterns of incidence for non-Hodgkin's lymphoma, as has been done in some previous studies, but there were too few cases in these states (e.g. less than 15 cases for our base case from 1980 to 1989) for such analyses to be meaningful.

### Study population and data

ALL and CNS incidence data were obtained from the SEER database (SEER, 2001). The SEER Program was begun in 1973 to collect, analyse and disseminate data useful in prevention, diagnosis and treatment of cancer. It was designed as a nonrandom (to capture ethnic diversity), population-based sample of about 10% of the US population. Records of individual cases are available to researchers, containing basic demographics, county of residence at diagnosis, state of birth, and basic diagnosis and treatment information. We considered all SEER registries with data dating back to the 1970s. We excluded those that were primarily urban and those that did not have any strongly rural counties (i.e. <100 persons/mi^2^) leaving only Iowa, New Mexico and Utah. SEER provides population at risk data as annual age specific, county level population count estimates for 1973–1999 from interpolations of the decennial censes conducted by the US Census Bureau, updated with information on births, deaths, internal migration and international migration (SEER, 1997). For age-specific incidence rates for a specific period, we divided the total number of cases within an age range in a county during that period by the sum of the population estimates for that same age range. We also used these SEER population data, aggregated over all ages, to assess the magnitude of county specific changes in population.

To balance the need for a time period long enough to accrue a sufficient number of cases to be statistically reliable but short enough that population and migration patterns would not have varied greatly, we used a 10-year period. In total, 10-year periods also coincide with the frequency of census data collection. As the SEER data were available from 1973 to 1999, we used the three time periods to best coincide with the census data: 1973–1979, 1980–1989 and 1990–1999.

Since ALL is most prevalent in Caucasian children, we limited our analyses to Caucasians. Inclusion of other races/ethnicities would likely dilute any observed effect while not providing sufficient numbers of cases for race/ethnicity-specific inferences.

Initially, we assessed the heterogeneity of the data among states and over decades to determine whether to pool the data or conduct separate analyses. First, we selected a single set of values for the variables of interest, specifying age, race/ethnicity, latency, birth location and ruralness. We call this our base, or reference, case and use it as the basis of comparison in our analyses. For the time period of observation for our base case, we chose the middle decade, 1980–1989. We also considered time periods of 1973–1979 and 1990–1999 as well as the entire 1973–1999 period. For the age of subjects, we chose ages 0–4 as many previous studies have found the population mixing effect most evident among these children. We also considered age groups of 5–9, 10–14, 15–19 and combined ages 0–14. Some previous studies included a latency adjustment for the development of ALL (Koushik *et al*, 2001). We used a latency of 1 year for our base case but also considered 0 and 2 years as latency adjustments. This was implemented for 1-year latency by estimating the population change from the year before the start of the study period (i.e. 1979 for the base case) to the year before the end of the study period (i.e. 1988 for the base case). A total of 2-year latency was estimated similarly by using population estimates from 2 years before the start and end of the cancer incidence period. For pre-1973 population estimates, not available in the SEER data, we used the 1973 values.

Under the Population Mixing Hypothesis, children who live in rural, isolated communities that are subject to a large, urban population influx are at increased risk of ALL (Kinlen, 2000b). Therefore, for our base case, we limited eligible subjects to those born and diagnosed in the same state (SEER provides county of residence at diagnosis but only state of residence at birth). We compared those results to those including all cases diagnosed in the selected states irrespective of place of birth.

As required by the hypothesis, we limited our analyses to rural counties (<100 persons mi^−2^) based on the population density at the start of the interval under study, such as the 1980 population density for the base case decade, 1980–1989. This is a rather strict definition of a rural county, but given the large size of counties, we believe this restriction lowered the likelihood of including as rural a county with a small urban area. We compared these results to the results excluding counties with population densities greater than 500 persons mi^−2^ and to the results obtained including all counties. The maximum county population density observed in these data was less than 850 persons mi^−2^. Concern about isolation may not be adequately addressed by limiting analyses to rural counties in that residents near a population center may make frequent trips into the city (Alexander *et al*, 1990). Since we did not have information about people's travels, we conducted an analysis in which we removed all counties that were adjacent to a county that was not rural.

To assess the sensitivity of the results to our selection of these specific values, we conducted additional analyses in which we changed the value of one variable at a time and compared the results to those of the base case analysis.

### Statistical methods

Separately for ALL and CNS cancers, we compared for specified time periods the incidence rates in counties that experienced a decline in total population or no change to counties that experienced >0% to 10% change in total population, >10 to 20% change and >20% change, as did Koushik *et al*. (2001), using logistic regression analysis of individual case data. Given the rarity of these diseases, we repeated some of these analyses using Poisson regression and obtained identical results. We used SAS v.8 for all analyses but also crosschecked a few runs using SPSS v. 11.

In a preliminary analysis to assess the heterogeneity over decades and across states, we conducted a series of logistic regressions for the entire study period, 1973–1999. We first calculated the odds ratios (ORs) for the different categories of population mixing, and then separately added dummy variables for decade of study, for state of residence, and both decade and state together. We also considered effect modification (i.e. interactions).

For our main analyses, we conducted three sets of regressions. First, we ran logistic regressions in which a dummy coded categorical variable reflecting the four classes of population change was forced into the model as an independent predictor of leukaemia incidence. Second, to assess the impact of a more crude assessment of population change, we ran separate logistic regressions forcing in dichotomous variables for population change (none or negative *vs* positive) and rural character (less than 100 persons mi^−2^ *vs* equal to or more than 100 persons mi^−2^, and for three categories with cutpoints of 100 and 500 persons mi^−2^ respectively). Third, to assess possible confounding and effect modification by rural environment, we ran logistic regressions in which a categorical variable reflecting the four classes of population change and a categorical variable reflecting rural character and their interaction terms as candidate predictor variables. For this latter assessment, we used a forward stepwise algorithm with *P*-values 0.15 and 0.20 for variable entry and exit criteria for the main variables and 0.05 and 0.10 for the interaction terms (Hosmer and Lemeshow, 2000). In addition, to assess the sensitivity of our results to our chosen parameter values, we performed regressions using the alternative values described above.

We also ran regressions with population change as a continuous rather than categorical variable but do not focus on these because small data sets such as ours may be unduly sensitive to outliers and do not adequately capture nonlinear or nonmonotonic relationships, making interpretation more difficult.

## RESULTS

Table 1

**Table 1:** An evaluation of temporal and geographic heterogeneity

								*Deviance*
								*−2ln* *L*
*Intercept* only								3109.66
*Intercept* plus *population change*								
	Percentage change in population size							
	>0–10%	>10–20%	>20%					
ALL	1.5 (0.9–2.3)^a^	2.0 (1.3–3.3)	2.0 (1.3–3.1)					3094.21 (*P*<0.002)
								
*Intercept, population change* plus *state*								
	Percentage change in population size			New Mexico	Utah			
	>0–10%	>10–20%	>20%					
ALL	1.5 (0.9–2.3)	2.0 (1.2–3.4)	2.1 (1.3–3.4)	1.1 (0.7–1.7)	0.8 (0.5–1.3)			3091.59 (*P*>0.26)
								
*Intercept, population change* plus *decade*^b^								
	Percentage change in population size			1973–1979	1990–1999			
	>0–10%	>10–20%	>20%					
ALL	1.7 (1.0–2.9)	2.0 (1.2–3.4)	2.3 (1.4–3.8)	0.6 (0.4–1.0)	0.4 (0.3–0.6)			3073.38 (*P*<0.001)
								
*Intercept, population change, decade* plus *state*								
	Percentage change in population size			1973–1979	1990–1999	New Mexico	Utah	
	>0–10%	>10–20%	>20%					
ALL	1.8 (1.0–3.2)	2.1 (1.1–4.2)	2.6 (1.4–5.0)	0.7 (0.4–1.1)	0.4 (0.3–0.6)	1.0 (0.6–1.6)	0.7 (0.4–1.2)	3070.46 (*P*>0.23)

a OR (95% CI).

b Addition of terms for effect modification did not provide a statistically significant improvement of the model (i.e. *P*>0.05). Referent categories: ⩽0% change in population size, Iowa, 1980–1989 (see text for details).

is a summary of the results of the analysis of heterogeneity. The column on the far right shows the deviance (–2 in likelihood) for assessing the model fit. Statistically significant decreases of this measure indicate a statistically significant improvement in the model. Addition of the population change variable, our principal hypothesis, yielded a model that is statistically superior to the model with no variables. Addition of the state of residence to the model with population change only did not improve the model significantly, but addition of decade of analysis only did improve the model significantly. Addition of state of residence to the model with population change and decade still did not improve the model significantly. There was no significant effect modification of population change by decade. Point estimates changed only slightly with addition of state and/or decade. In sum, there was heterogeneity in ALL rates across magnitude of population change and independently by decade, but not across state of residence, and there was no effect modification between population change and decade. Therefore, analyses for population mixing were conducted separately for each decade but pooled for all three states.

Table 2

**Table 2:** Risk for incidence of disease versus change in population size^a^

		**Percentage change in population size**			**Number of cases**	
		**>0–10%**	**>10–20%**	**>20%**	**In data set**	**In analysis**
*Base case*^b^						
	ALL	1.9 (1.0–3.6)^c^	2.1 (1.1–3.8)	2.6 (1.5–4.6)	(141)	(82)
	CNS	1.2 (0.6–2.4)	1.3 (0.7–2.4)	0.7 (0.3–1.7)	(113)	(64)
						
*Exclude if adjacent to a nonrural county*						
	ALL	2.6 (1.1–6.4)	2.4 (1.0–5.5)	4.3 (2.0–9.7)	(42)	(42)
	CNS	1.0 (0.3–3.0)	1.6 (0.7–3.6)	1.1 (0.4–3.2)	(32)	(32)
						
*Age range*						
5–9	ALL	1.9 (0.7–5.3)	2.4 (1.0–6.0)	––	(62)	(25)
	CNS	0.8 (0.3–2.5)	1.6 (0.7–3.6)	0.4 (0.1–1.8)	(62)	(31)
10–14	ALL	2.1 (0.6–7.1)	1.0 (0.2–4.7)	1.1 (0.2–5.1)	(43)	(15)
	CNS	1.5 (0.5–4.2)	1.1 (0.4–3.5)	1.2 (0.4–3.8)	(42)	(25)
15–19	ALL	––	0.3 (0.0–2.2)	0.6 (0.1–2.8)	(33)	(16)
	CNS	3.2 (1.0–10.5)	1.2 (0.2–6.1)	3.3 (1.0–11.0)	(38)	(18)
0–14	ALL	2.0 (1.2–3.2)	2.0 (1.3–3.3)	1.8 (1.1–3.0)	(246)	(122)
	CNS	1.2 (0.7–1.9)	1.4 (0.9–2.2)	0.8 (0.4–1.4)	(217)	(120)
						
*Latency*						
0 years	ALL	2.1 (1.2–3.9)	1.9 (1.1–3.5)	2.5 (1.4–4.5)	(141)	(82)
	CNS	1.3 (0.6–2.5)	1.5 (0.8–2.8)	0.7 (0.3–1.9)	(113)	(64)
2 years	ALL	2.1 (1.1–4.2)	2.1 (1.2–3.8)	1.8 (1.1–3.2)	(141)	(89)
	CNS	1.5 (0.8–3.2)	1.6 (0.9–2.8)	0.7 (0.4–1.3)	(113)	(70)
						
*Birth location*						
Any state	ALL	1.6 (1.1–2.5)	1.1 (0.6–1.7)	1.6 (1.0–2.5)	(270)	(144)
	CNS	1.3 (0.7–2.2)	1.5 (0.9–2.5)	0.9 (0.5–1.7)	(171)	(95)
						
*Urban definition*						
>500/mi^2^	ALL	1.5 (0.8–2.6)	1.8 (1.2–2.9)	1.6 (1.0–2.6)	(141)	(119)
	CNS	1.2 (0.7–2.0)	0.8 (0.4–1.4)	0.9 (0.5–1.4)	(113)	(99)
None	ALL	1.5 (0.9–2.5)	1.4 (0.9–2.1)	1.6 (1.0–2.6)	(141)	(141)
	CNS	1.0 (0.6–1.7)	0.7 (0.4–1.1)	0.9 (0.5–1.4)	(113)	(113)
						
*Year range*						
1990–1999	ALL	1.2 (0.4–3.2)	1.5 (0.5–4.6)	1.6 (0.6–4.1)	(78)	(36)
	CNS	2.8 (0.8–9.7)	3.5 (0.9–13.3)	2.6 (0.7–9.4)	(76)	(34)

a Referent category of population change is ⩽0% change.

b Base case conditions: 1980–1989; ages 0–4; Caucasian; latency 1 year; born and diagnosed in same state; excludes urban areas (>100 people mi^−2^).

c OR (95% CI).

shows the results of our main analyses using the categorical variable for population change. The first column identifies the specific analysis reported in the following two adjacent rows. The second column shows the disease reported in that row, either ALL or CNS cancers. The third through fifth columns report the ORs with 95% confidence intervals (CIs) from the logistic regressions for categories of population change. The sixth column displays the combined number of cases available for that analysis (i.e. the specified age range, year range, latency, birth location). The seventh column shows the number of cases included in the analysis after excluding the more urban counties.

With respect to the most notable findings, for the base case, the results obtained were consistent with the Population Mixing Hypothesis. The relative risk of ALL increased as a function of population change from 1.9 for up to 10% increase as compared with no increase or a decline, to 2.1 for >10% to 20% increase, to 2.6 for >20% increase, with an apparent dose–response and the lower 95% CIs are >1.0. The comparison to CNS cancer incidence for the same base case showed substantially smaller elevations of risk for the first two of the three categories of population change and decreasing risk for the third category, and none of the 95% CIs excluded 1.0. The rate ratios for each category comparing ALL to CNS, if calculated, would have exceeded 1.0. In our analyses restricted to more isolated counties (removing those adjacent to a nonrural county), the pattern in ALL was even more pronounced with ORs from 2.4 to 4.3, and those for CNS barely changed. However, due to the reduced sample size, CIs were larger and thus results were less reliable.

When we varied the age range of children, the number of cases was relatively small in each 5-year age group resulting in somewhat inconsistent patterns and limiting interpretability. The pattern for 0–14 years showed statistically significant elevated rates for all population change categories for ALL. The results for CNS cancers were less clear with one OR below 1.0, and none statistically significant. These observations are weakly consistent with the Population Mixing Hypothesis and consistent with work of others (Kinlen, 1988; Kinlen *et al*, 1990,^1995^,^2002^; Kinlen and Stiller, 1993; Kinlen and Balkwill, 2001; Kinlen and Bramald, 2001; Koushik *et al*, 2001). Evaluation of latency periods resulted in only minor changes in results from the base case, as expected.

When we relaxed the restriction that cases had to be born in the same state as diagnosed the results were similar but weaker than the base case for ALL but similar to the base case for CNS cancers.

We excluded urban counties using two definitions of urban. First, we made the definition of rural less restrictive by excluding only counties with more than 500 persons mi^−2^. For ALL, this resulted in a similar but weaker pattern to that observed in the base case, with a dose–response and two 95% CIs >1.0; the CNS cancer pattern did not change appreciably. Second, we removed the population density restriction entirely and included all counties in our analyses producing even weaker results but still consistent with the Population Mixing Hypothesis.

We conducted analyses similar to the base case for the periods 1973–1979 and 1990–1999. For 1973–1979 (results not shown), there were so few cases, and so many counties had decreasing populations, that ORs could not be calculated. For the 1990–1999 period, all ORs for ALL and CNS exceeded 1.0. The pattern observed for CNS cancers showed larger relative risks than those for ALL, although the sample size for each was considerably smaller than our base case and the 95% CIs were very large, making results unreliable. These results do not provide evidence supporting or contradicting our hypothesis.

We also conducted analyses using dichotomous predictor variables for population change (>0%) and rural environment (<100 people per square mile) for 1980–1989 and 1990–1999. First, for population change, which compared no change or negative change in total population to any increase in total population, the effect in the 1980–1989 period was strong (OR=1.5; 95% CI 1.0–2.2) and was larger than that for CNS cancers (OR=0.8; 95% CI 0.5–1.2). For the 1990–1999 period, the effect was elevated for ALL analysis but smaller than that for CNS cancers. Using rural environment as a possible predictor of disease risk gave slightly elevated ORs for both ALL (OR=1.5; 95% CI 1.1–2.1) and CNS (OR=1.4; 95% CI 1.0–2.4) cancer for 1980–1989, with both just statistically significant. Results for 1990–1999 yielded ORs of 1.0 for both ALL and CNS. Using a more relaxed definition of rural residence (<500 people per square mile) gave similar results for ALL (OR=1.3; 95% CI 0.8–2.1) and slightly stronger and marginally significant results for CNS (OR=1.7; 95% CI 1.0–3.0). These results are weakly consistent with the hypothesis, showing that population change can result in increased risk but that having low population density alone was not sufficient.

We also conducted analyses investigating effect modification between categorical population change and dichotomous strict ruralness, using stepwise inclusion criteria to select those variables that best predicted the outcome (results not shown). For the base case for ALL we found increasing risk with increasing population change and an effect for living in a rural county but no effect modification. For the base case for CNS cancers, only the rural county variable was selected and its 95% CI did not exclude 1.0. By definition, no effect modification was found. Similar results were found for all three categories or degree of ruralness (or population density). These results taken together suggest that the increases in population size were associated with increased risk of ALL among young children as compared to CNS cancers, were confounded by rural status, but the effect was not modified by the rural status. Conversely, the effect of a rural residence on ALL risk was slightly enhanced when adjusted for population change.

Finally, we analysed these data using population change and population density as continuous variables. For ALL, the OR for the entire data set was 1.013 for a one percent population change (95% CI 1.004–1.021), and for rural residence only it was 1.015 for a 1% population change (95% CI 1.007–1.023). For CNS, the OR for the entire data set was 0.995 for a 1% population change (95% CI 0.983–1.007), and for the rural residence only it was 0.998 for a 1% population change (95% CI 0.985–1.011). Inclusion of population density in the model did not change the point estimates, so there was no observed confounding. For population density only, the ORs were 0.999 for both ALL and CNS. Again, due to the unusual distribution of the data, the small sample size and the occurrence of outliers and nonmonotonic relationships, these results are of limited interpretability. Nonetheless, the data showed a statistically significant relationship between population change and ALL but not for CNS, and did not show a statistically significant relationship between population density and either ALL or CNS.

## DISCUSSION

The results obtained in this study are intriguing and many are consistent with the Population Mixing Hypothesis. However, they are limited due to the nature of the data, the small number of cases and the types of analyses employed. At the outset of this study, we recognised that data limitations would preclude confirmation of the hypothesis but might provide support. On the other hand, we believed that results inconsistent with the hypothesis would raise serious questions about its possible validity.

The basic hypothesis is that children in rural isolated communities into which there has been a rapid influx of people are at increased risk of leukaemia as a result of a (undetected) viral epidemic. In contrast, areas with a higher population density would be more likely to have established herd immunity, thereby preventing the relevant epidemics and subsequent leukaemias. Kinlen and colleagues have studied childhood leukaemia in a wide range of situations in which isolated populations were exposed to a large influx of outsiders or other sources of infectious agents (Kinlen *et al*, 1990,^1993^,^1995^,^2002^; Kinlen and Stiller, 1993; Kinlen and John, 1994; Kinlen and Petridou, 1995; Kinlen, 1993,^1997^,2000a,2000b; Kinlen and Balkwill, 2001; Kinlen and Bramald, 2001). Other investigators have undertaken similar investigations in other situations and in other countries and obtained largely similar results (Langford, 1991; Roman *et al*, 1994; Dockerty *et al*, 1996; Petridou *et al*, 1996; Stiller and Boyle, 1996; Alexander *et al*, 1997,1998a; Dickinson and Parker, 1999; Fear *et al*, 1999; Birch *et al*, 2000; Koushik *et al*, 2001; Boutou *et al*, 2002; Dickinson *et al*, 2002a,2002b; Dickinson and Parker, 2002; Parslow *et al*, 2002). The effect is seen most strongly for ALL at ages 0–4 and in the most rural areas (Kinlen *et al*, 1990,^1993^,^1995^,^2002^; Stiller and Boyle, 1996; Alexander *et al*, 1997; Koushik *et al*, 2001; Kinlen and Balkwill, 2001; Kinlen and Bramald, 2001). Properties of rural areas include low population density, relative isolation, limited commuting or visitation from other areas, etc, although specific definitions vary by county. Others have explored the use of population density as the predictor of high cancer rates (rather than population mixing) with somewhat mixed results (Alexander *et al*, 1990,1998b,^1999^; Ross *et al*, 1999). One recent paper provided somewhat contradictory results, showing that increased risk of childhood leukaemia associated with inward migration was more marked in urban than rural areas (Dickinson *et al*, 2002a).

We used SEER data to evaluate the Population Mixing Hypothesis for several reasons: these data are of high quality, have high reporting rates, are readily available to researchers and include a substantial portion of the US population. SEER data, however, also have several limitations. First, the geographic resolution is too broad. Location of diagnosis is provided at the county level. Regions can vary from urban to rural within a county and this variation is not reported. This is in part why we limited our analyses to very rural counties. Second, information on residence history is unavailable. Only state of birth and county of diagnosis are available. We chose to assume that those born and diagnosed in the same state had lived in the same county between birth and diagnosis. This will not always be true but we have no data with which to quantify the number of mistakes made by this assumption. We believe that this effect is likely to be nondifferential between ALL and CNS cancer cases although deviation from this assumption could weaken the findings.

We considered using mortality data that also have high reporting rates, are readily available and include the entire US population, but felt that they were potentially biased with respect to the Population Mixing Hypothesis because survival for childhood leukaemia is about 80 percent in our principal study period (Smith *et al*, 1999).

We examined whether limiting the analyses to isolated, rural areas would result in a stronger effect and it did although the sample size was small. Ideally, we would have used migration or travel to urban areas data to adjust for isolation, but no such data are readily available.

Owing to our exclusions, and the rarity of the ALL, all our sample sizes were fairly small. Even though some of the ORs were statistically significant, the reliability of the specific estimates is low.

Despite all of these limitations, our data are consistent with the Population Mixing Hypothesis. The base case showed a relatively strong and consistent pattern. We can explain deviations in the sensitivity analyses at least from a *post hoc* perspective although we recognise the limitations of such after the fact assessments. We did not see the same pattern in the 1990–1999 data, but in these years sample size is even smaller, mobility and travel had increased relative to the 1980s, and counties that were isolated in the 1980s may no longer have been so. For this time period, the biggest change was in the pattern of CNS cancers, although the CIs were unusually large, calling into question the validity of the ORs.

The failure to find consistent patterns in different age children may suggest that the older children had been exposed to infectious agents earlier in life and may have developed immunity to the putative viral agent(s). In addition, the numbers in each stratum were much smaller than for those 0–4 years. The hypothesised effect is seen in the 0–4 and 0–14 groupings, although for ALL the latter is dominated by those at 0–4. The length of latency did not affect the pattern, cases born out of state did not show the effect and residence in less rural counties diluted but did not remove the observed effect. The absence of effect modification runs counter to one previous study (Koushik *et al*, 2001), but again may reflect small sample size effects.
